# Perceptual Coupling Based on Depth and Motion Cues in Stereovision-Impaired Subjects

**DOI:** 10.1177/0301006620952058

**Published:** 2020-09-09

**Authors:** Laurens A. M. H. Kirkels, Reinder Dorman, Richard J. A. van Wezel

**Affiliations:** Donders Institute for Brain, Cognition and Behaviour, Department of Biophysisc, Radboud University, The Netherlands; Swammerdam Institute for Life Sciences, University of Amsterdam, The Netherlands; Donders Institute for Brain, Cognition and Behaviour, Department of Biophysisc, Radboud University, The Netherlands;; TechMed Centre, Department of Biomedical Signals and Systems, University of Twente, The Netherlands

**Keywords:** stereopsis, depth, three-dimensional perception, binocular vision, bistability, perception

## Abstract

When an object is partially occluded, the different parts of the object have to be perceptually coupled. Cues that can be used for perceptual coupling are, for instance, depth ordering and visual motion information. In subjects with impaired stereovision, the brain is less able to use stereoscopic depth cues, making them more reliant on other cues. Therefore, our hypothesis is that stereovision-impaired subjects have stronger motion coupling than stereoscopic subjects. We compared perceptual coupling in 8 stereoscopic and 10 stereovision-impaired subjects, using random moving dot patterns that defined an ambiguous rotating cylinder and a coaxially presented nonambiguous half cylinder. Our results show that, whereas stereoscopic subjects exhibit significant coupling in the far plane, stereovision-impaired subjects show no coupling and under our conditions also no stronger motion coupling than stereoscopic subjects.

In vision, the brain uses spatial and temporal context to disambiguate visual information such as stereoscopic disparity, occlusion, shading or speed, and direction of motion information. The role of motion in the inference of three-dimensional structure from context can be illustrated with the kinetic depth effect (Miles, 1931; Wallach & O’Connell, 1953), also known as structure from motion (SFM; Andersen & Bradley, 1998; Nawrot & Blake, 1989; Ramachandran et al., 1988; Treue et al., 1991). An example of this effect is a transparently rotating cylinder created from two random-dot fields that overlap and move in opposite directions (Andersen & Bradley, 1998; Nawrot & Blake, 1989; Treue et al., 1991). Without obvious depth ordering, the perceived rotation direction of the cylinder can switch back-and-forth unpredictably, while the physical properties of the stimulus have not changed (for reviews, see Blake & Logothetis, 2002; Leopold & Logothetis, 1999). Adding context or stereo depth cues, however, could lead away from bistability and toward one (almost) stable percept.

Another example of disambiguation of SFM stimuli is through perceptual coupling of a set of multiple coaxially rotating ambiguous SFM stimuli (Eby et al., 1989; Freeman & Driver, 2006; Gillam, 1972; Grossmann & Dobbins, 2003). Although the timing of percept switching is still unpredictable, the perceptually coupled stimuli switch their direction synchronously. Perceptual coupling is largest for two ambiguous stimuli and can be affected by disambiguating the context, for instance, by changing the alignment of the axes (Bonneh & Gepshtein, 2001), by adding a luminance gradient and disparity cues (Freeman & Driver, 2006), or by small interstimulus separations (Gilroy & Blake, 2004). Interestingly, although full disparity does not abolish the coupling, a maximal luminance gradient between the two stimuli results in a strong reduction or abolishment of the coupling (Freeman & Driver, 2006). From this, it was proposed that both surface layers of the cylinders need to be present for perceptual coupling (Freeman & Driver, 2006). This requirement was challenged in a later study by Klink et al. (2009), where it was shown that it was possible to perceptually couple an ambiguous full cylinder with a coaxially presented disparity-defined half cylinder. Their results show that only a half cylinder defined by disparity for the far side produced strong coupling.

In case of stereovision impairments or even stereoblindness, subjects are unable to process disparity information correctly and do not perceive stereo depth. This impairment can have different causes such as blindness in one eye, amblyopia (a lazy eye; Levi, 2006), or strabismus (eye misalignment; Lorenz, 2002). Causes in the underlying mechanisms of impaired stereovision could be found in a lack of binocular correlation during the critical period for development of stereopsis as a consequence of squint (Hubel & Wiesel, 1965), vergence anomaly (Jones, 1977), or congenital lack of binocular neurons (Cool & Crawford, 1972; Hubel & Wiesel, 1971). Another origin of impaired stereovision could be sought at the optic chiasm, for instance, in case of achiasmatic syndrome (Apkarian et al., 1995) or albinism (Hoffmann et al., 2003). Other subjects might have difficulties in judging the direction of the disparity, indicating there are multiple classes of disparity detectors resulting in stereo anomalies and that stereoblindness results from the absence or dysfunction of all disparity categories (Richards, 1970; but also see Dorman & van Ee, 2017). Yet, it sometimes is possible to integrate motion and disparity for stereo anomalous subjects that still have a part of the disparity spectrum that processes correctly (Ee, 2003). Stereovision-impaired subjects, on the other hand, do not have a proper functioning part of their disparity spectrum and are therefore regarded as being unable to perceive stereoscopic depth. Compared to subjects with normal stereopsis, stereovision-impaired subjects (in case of congenital defect) developed vision under different conditions, resulting in partial or complete loss of stereovision. Although stereovision-impaired subjects have little or no access to stereoscopic disparity information, due to their lack of binocular neurons (Blake & Cormack, 1979; Blake & Hirsch, 1975; M. L. Crawford et al., 1984; M. L. J. Crawford et al., 1996; Hubel & Wiesel, 1965), they are still receptive to motion cues. Earlier research suggested that these motion cues can be essential for depth information (Johnston et al., 1993, 1994; Young et al., 1993). With processing of motion cues still intact, stereovision-impaired subjects might have reorganized their depth-related networks via Hebbian learning. If indeed occlusion-related coupling also relies on motion coupling, this would be reflected in performances of stereovision-impaired subjects in the tests with the transparent rotating (half) cylinders. We therefore hypothesize that stereovision-impaired subjects use compensatory strong motion coupling in deciding about depth.

We tested stereoscopic and stereovision-impaired subjects for perceptual coupling by presenting one disparity-defined (near or far) transparent half cylinder rotating coaxially to a full ambiguous transparent cylinder. Subjects were asked to report motion direction of the front plane of the full ambiguous cylinder, in order to determine the extent of motion coupling.

## Materials and Methods

### Subjects

Subjects (*n* = 19) were recruited from the student and staff population (11 females/8 males) of the Radboud University with an age ranging from 20 to 47 years (average 27.4 ± 7.2). Stereovision-impaired subjects were recruited based on their verbal report of inability to see stereoscopic depth. In addition, subjects provided details about their visual anomalies from early childhood onwards. Three subjects suffered from amblyopia in childhood, which was corrected. Two other subjects experienced strabismus, one of which had his eyes rectified and the other subject only experienced a mild strabismus. Subjects signed an informed consent and privacy statement. All subjects were first subjugated to a rough screening of stereoscopic depth perception, by passing or failing the Stereo Fly Test. Note that the Stereo Fly Test can be passed based on nonstereoscopic cues (Chopin et al., 2019; De La Cruz et al., 2016). Furthermore, we tested their binocular vision with a binocular rivalry stimulus using red/green anaglyph glasses and a superimposed house/face stimulus (Tong et al., 1998). This test provides information on eye dominance and binocular summation. We defined a normal response to binocular rivalry stimuli as a single-eye percept at one time, switching to the other eye in a several of seconds in an irregular fashion with only sparse episodes of mixed percepts (Blake & Logothetis, 2002). Stereovision-impaired subjects with amblyopia would present with a nonrivaling percept, perceiving only the input from the dominant eye. Finally, a quantitative experiment was performed to test depth perception using the experimental paradigm described later. Based on the results of this baseline test, we divided the group of subjects in stereoscopic and stereovision-impaired. One subject, that passed the Stereo Fly Test and did not report impaired stereovision, only partially completed the experiment. Because of an incomplete dataset, we removed this subject from our study and continued with 18 subjects.

### Stimulus

**Figure 1.**
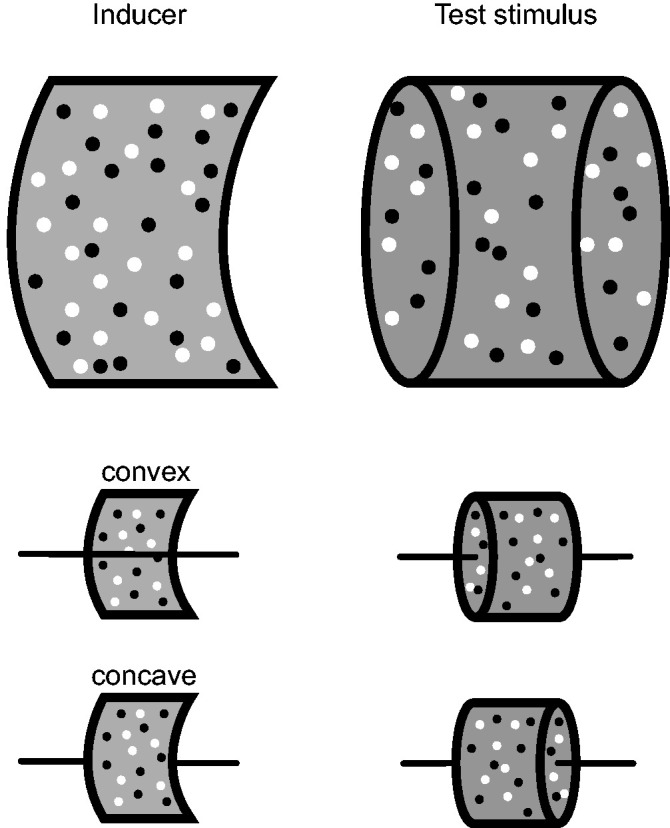
Stimulus. Schematic drawing of the inducer (half cylinder) and test stimulus (full cylinder) that are defined by moving random dot patterns consisting of black and white dots on a gray background. The position of each dot was randomly generated on the *X* and *Y* plane of each cylinder half. The inducer stimulus is defined by stereoscopic disparity as a backplane (*far*) or foreplane (*near*) of a half cylinder, respectively, referred to as convex and concave. The test stimulus, consisting of two transparently oppositely moving patterns, is perceived as a full cylinder that rotates upwards or downwards and this direction of rotation is ambiguous.

We used a kinetic depth stimulus (Figure 1) as described in Klink et al. (2009). It consisted of a red fixation cross of 0.25° in the middle of the visual scene. Two random dot SFM cylinders were presented on the left and right side of the cross. The left cylinder consisted of only one moving layer, giving the impression of a half cylinder, which could be either convex (*near*) or concave (*far*). The right cylinder consisted of two layers, giving the impression of a full cylinder. Both cylinders were 3 × 3 visual degrees, separated by 0.5 visual degrees, and consisted of black and white dots, respectively 0 cd/m^2^ and 28 cd/m^2^, on a gray background of 14 cd/m^2^. The dot size was 0.11 visual degree and the dot density for both layers was 12 dots/deg^2^, giving 108 dots in the full cylinder. There was no overlap of dots in the single fields that were used. In displaying two fields of dots transparently, the position of each dot was randomly generated on the *X* and *Y* plane of each cylinder half. Overlap was therefore possible. However, since generally, the stimuli generated a proper three-dimensional percept of a convex/concave cylinder, we assume that potential overlap between two white and black dots in the same plane did not cause any difficulties. The cylinder made a full turn (360°) in 3 seconds, therefore turning at an angular velocity of 120 deg/s. This resulted, on display surface, in a dot speed in visual angle of about 2 deg/s in either upwards or downwards direction, randomly selected per trial. Stereoscopic depth was introduced by means of horizontal disparity gradients, with the maximum disparity being a perfect round cylinder with a diameter of 3°, which translates back to 5.7 arcmin on the display surface. Stimuli were presented for 1 second, followed by a 1.5 second interval until the next stimulus. Subject responses were given in the off-period.

### Apparatus

Visual stimuli were generated on a computer using MATLAB 2012a (The MathWorks, Natick, MA, 2012), using the PsychToolbox (Brainard, 1997; Pelli, 1997). Stimuli were presented on a 22-inch CRT monitor, with a refresh rate of 100 Hz. A gamma correction was used to ensure linearization of stimulus luminance. Observers viewed the stimulus from 100 cm through a mirror stereoscope, positioned at the middle of the monitor. The angle of binocular convergence was set by the individual subjects before the start of each experiment, by adjusting the mirror stereoscope such that two binocularly presented crosses exactly overlapped. The experiments took place in a darkened room. Responses were captured using a standard USB keyboard.

### Paradigm

The experiment consisted of two training sessions, to familiarize the subject with the task and the stimulus, and two experimental sessions. In the first training session, the subject was asked to report the shape of the left, half cylinder; either convex or concave. In this session, stereoscopic cues were used to give the left half cylinder depth. A feedback flash was given in either green or red to tell the subject whether the given answer was correct or not. In case of 0 arcmin disparity, the flash was always green (since the stimulus is flat, there is no right or wrong). In the second training session, the subjects were asked to report the direction of movement of the front layer of the right full cylinder. In this session, the right cylinder was given stereoscopic depth cues. Both training sessions consisted of only five repetitions and 0, 2.3, and 4.6 arcmin disparity. When a subject had trouble with the shapes of the tasks, the training was extended.

The first experiment was a baseline experiment. In this experiment, no feedback was given; subjects reported if the left half cylinder was convex or concave. The stimulus paradigm consisted of 20 repeats, with disparity of 0, 2.3, and 4.6 arcmin, in both concave and convex fashion. These disparities were chosen based on the results from pilot experiments. The task was divided in blocks of 120 trials, lasting 5 minutes each. Subjects were allowed to take as many breaks as they wanted to reduce fatigue and retain the ability to focus.

The second experiment was the coupling experiment. Subjects reported the moving direction of the frontal plane of the right full cylinder, which was now completely ambiguous (0 arcmin disparity). The left cylinder was given stereoscopic depth cues in 4.6 arcmin disparity, either convex or concave. The experiment was performed in two blocks with either the convex or concave left cylinder.

### Statistical Analysis

**Figure 2.**
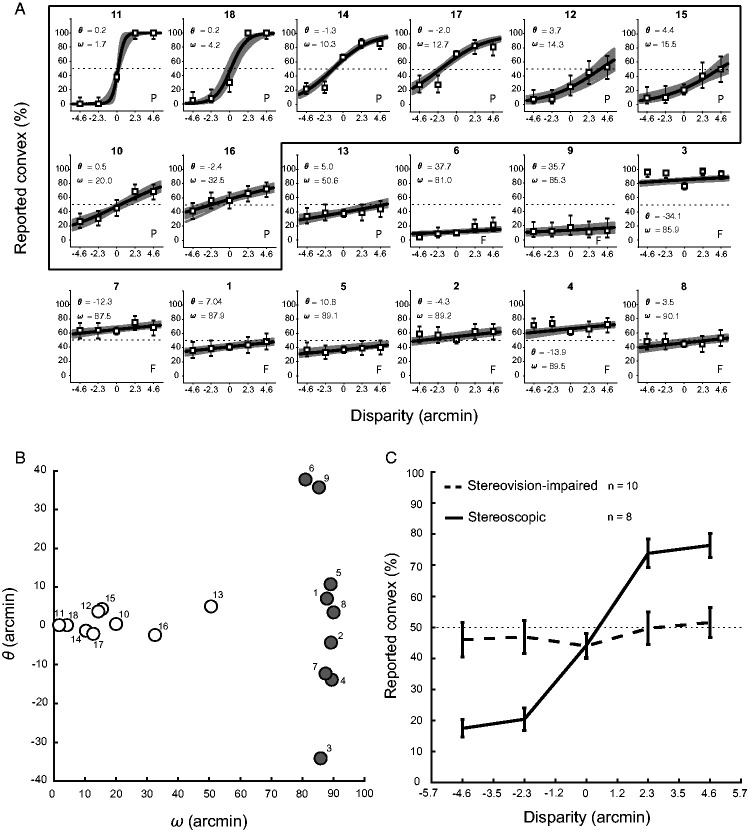
Convexity. (A) Reports on convexity for half cylinders with different degrees of disparity in arcmin (convex = positive values and concave = negative values) for individual subjects (*N *=* *18). Outline marks separation of stereoscopic (inside) versus stereovision-impaired (outside) subjects. Thick solid black line represents subject’s predicted psychometric curve based on individual data (gray thin lines). Thin dashed line represents 50% probability, error bars indicate *SD*. θ indicates disparity where subjects has 50% probability of reporting cylinders with positive disparity as convex (correct), ω reflects disparity range for which probability of reporting convex varied from 0.05 to 0.95. P (pass) or F (fail) indicates subject’s performance in Stereo Fly Test. Subjects are sorted on ω. (B) Plot of θ against ω as deduced from individual psychometric curves in (A). Numbers in (A) and (B) correspond to individual subjects. Individuals who did not pass the Stereo Fly Test are indicated with gray circles. (C) Reports on convexity for stereoscopic (solid line, *n *=* *8) and stereovision-impaired (thick dashed line, *n *=* *10) subjects. Thin dashed line represents 50% chance, error bars indicate *SEM*.

To determine how the convexity percept depended on disparity, we fitted a logistic psychometric curve (Figure 2A):

(1) $$P(\text{convex})={\left(1+e^{\frac{-4.39}{\omega }\left(\text{disparity}-\theta \right)}\right)}^{-1}$$

with θ the disparity value where a subject has a 50% probability to report cylinders constructed of positive disparities as convex (correct) and ω (psychometric curve width) the disparity range for which the probability of reporting convex cylinders varied from 0.05 to 0.95. A small ω reflects a clear dependence of the convexity percept on disparity, whereas a large ω implies an inconsistent correlation between the convexity percept and disparity and indicates the subject is stereovision-impaired. A nonzero, nonnegligible θ reflects a bias in perceiving the convexity of cylinders; positive and negative θ indicate a bias in perceiving concave and convex cylinders, respectively.

The fit parameters were obtained by custom-built Matlab functions (see van de Rijt et al., 2019) applying Bayesian inference using a Markov Chain Monte Carlo technique implemented in a Gibbs sampler program through MatJags (Brooks & Gelman, 1998, for general principles; for parametrization, see Kuss et al., 2005). Posterior distributions of parameters ω and θ were obtained for each participant separately, but we also determined hierarchical group-level parameters.

To cluster the subjects into stereovision-impaired and stereoscopic groups, we determined whether the estimated steepness of the psychometric curve for each subject differed from a small, null-hypothesis value, as follows. First, we took the inverse of ω as a measure of the steepness of the psychometric curve. Ideally, the null-hypothesis value of the slope would be 0, but a steepness of 0 was an extremely unlikely value observed in the data. Therefore, we then estimated the smallest credible steepness observed in the data at the lower limit of the 95%-highest-density interval of the steepness, which was 0.0076 (for Subject 8). Finally, we calculated Bayes factors (Jeffreys, 1961) based on which we would decide whether a subject was stereovision-impaired or stereoscopic. The Bayes factors were determined via the Savage–Dickey method (Dickey, 1971; Wetzels et al., 2010).

(2) $${\text{BF}}_{10}=\frac{p(y|H_{1})}{p(y|H_{0})}$$

The Bayes factor (BF) BF_10_ indicates how more likely the observed data *y* is under the alternative hypothesis *H*_1_ than under the null hypothesis *H*_0_. For our baseline experiment, the null hypothesis is defined as *H*_0_: 1/ω = 0.0076, while the alternative hypothesis is defined as *H*_1_: 1/ω ≠ 0.0076. BFs > 3 were taken to reflect a credible (cf. significant) difference between the alternative and null hypothesis. In general, Bayes factors can be interpreted and classified as substantial (3 < BF_10_ < 10), strong (10 < BF_10_ < 30), very strong (30 < BF_10_ < 100), and decisive (BF_10_ > 100) evidence (Jeffreys, 1961) for the alternative hypothesis, indicating that a subject is stereoscopic. The reverse applies to the null hypothesis, with substantial (0.1 < BF_10_ < 0.3), strong (0.03 < BF_10_ < 0.1), very strong (0.01 BF_10_ < 0.03), and decisive (BF_10_ < 0.01) evidence for a subject being stereovision-impaired.

We statistically tested each group in the baseline experiment for an effect of disparity using a one-way analysis of variance (ANOVA). Furthermore, *t* tests were performed against chance per disparity. In the motion coupling experiment, we performed *t* tests for far and near against chance and between near and far condition. We performed these tests independently for both the stereoscopic and the stereovision-impaired group.

## Results

We designed a human psychophysical experiment to test our hypothesis that stereovision-impaired subjects more prominently use motion cues for perceptual coupling. To quantitatively determine the ability for stereoscopic vision, we tested whether our subjects were able to correctly report the left half cylinder to be convex or concave. The individual data were fitted with a logistic psychometric curve (see Methods for details) giving two variables, θ and ω. In this fitted psychometric curve, ω is the disparity range for which the probability of reporting cylinders as convex varied from 0.05 to 0.95. The disparity value where a subject has a 50% probability to report cylinders constructed of positive disparities as convex is represented by θ. Figure 2A shows psychometric curves for individual subjects sorted by ω (small to large). A large ω means that disparities have no influence on subjects’ percentage of reporting convex. An average of their convex reporting percentage would therefore indicate a bias for seeing either convex or concave. For subjects with a small ω, θ is a good indicator for their bias, because these subjects’ reporting is influenced by disparity. A positive θ reflects a bias in perceiving concave, while subjects with a negative θ are more biased toward perceiving convex cylinders. Setting out values of θ against ω, the total group of subjects seems to divide into two groups (Figure 2B).

We classified whether subjects were stereoscopic-impaired or not based on the steepness of the psychometric curve (taken as inverse of ω*;* see Methods for details). We found very strong (BF_10_ > 30 for subjects 10, 12, 14–17) to decisive (BF_10_ > 100 for subjects 11 and 18) evidence to regard eight subjects as being stereoscopic. The other subjects were considered as stereovision-impaired based on decisive (BF_10_ < 0.01 for Subjects 1–9) and substantial (BF_10_ < 0. 3 for Subject 13) evidence of having a negligible steepness of the psychometric curve. For subjects classified as stereovision-impaired θ values covered a very broad range (−40 to 40 arcmin). As explained earlier, this can be expected since disparity has no influence on stereovision-impaired subjects’ percentage of reporting convex. However, subjects classified as stereoscopic all appear to exhibit relatively small θ values (−2.4 to 4.4 arcmin), indicating only slight biases for perceiving either convex or concave.

Although Subject 13 passed the Stereo Fly Test (open circle), we allocated this individual to the stereovision-impaired group based on the substantial evidence from the analysis above. This classification resulted in a stereoscopic group size of 8 and a stereovision-impaired group size of 10. Figure 2C shows the average results of the two groups. Stereoscopic subjects were able to distinguish between stimuli differing in stereoscopic depth cues (solid line), whereas the other group of subjects (stereovision-impaired) was unable (dashed line). A one-way ANOVA revealed no significant effect of disparity for the stereovision-impaired group, *F*(4,45) = 0.14, *p *=* *.97. *T* tests revealed no significant deviation of the 50% (chance) line for any of the disparities in the stereovision-impaired group (−4.6 arcmin against chance: *t*(9) = −0.46, *p *=* *.66; −2.3 arcmin against chance: *t*(9) = −0.37, *p *=* *.72; 0 arcmin against chance: *t*(9) = −0.94, *p *=* *.37; 2.3 arcmin against chance: *t*(9) = −0.04, *p *=* *.97; 4.6 arcmin against chance: *t*(9) = 0.20, *p *=* *.84). For the stereoscopic subjects, the ANOVA showed a significant effect of disparity, *F*(4,35) = 17.82, *p * < * *10^−7^. Furthermore, all disparities except 0 arcmin appeared to be significantly different from chance in the stereoscopic group (−4.6 arcmin against chance: *t*(7) = −6.51, *p * < * *.001; −2.3 arcmin against chance: *t*(7) = −4.62, *p * < * *.01; 0 arcmin against chance: *t*(7) = −0.87, *p *=* *.41; 2.3 arcmin against chance: *t*(7) = 2.95, *p * < * *.05; 4.6 arcmin against chance: *t*(7) = 3.90, *p * < * *.01).

Next, we examined motion coupling in the stereoscopic and stereovision-impaired subjects. Subjects were asked to indicate the motion direction of the frontal plane of a full ambiguous cylinder presented coaxially to a half cylinder that was defined by disparity (Figure 1). The subjects’ responses were compared with the half cylinder’s motion direction. We predicted that stereoscopic subjects would show depth differentiated coupling (Freeman & Driver, 2006; Klink et al., 2009), whereas stereovision-impaired subjects would show motion coupling, regardless of stereoscopic depth.

**Figure 3.**
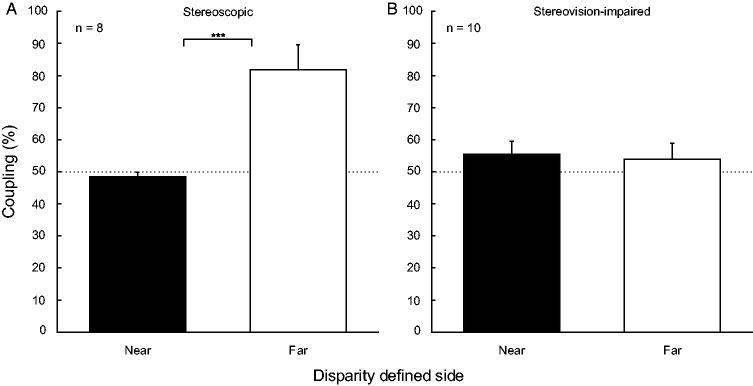
Motion Coupling for Stereoscopic and Stereovision-Impaired Subjects. Coupling of the half cylinder to the full cylinder in 4.6 arcmin disparity-defined near and far condition (solid black and white bars) for (A) stereoscopic (*n *=* *8) and (B) stereovision-impaired subjects (*n *=* *10). Percentage of coupling reflects *correct* reporting of the motion direction of the full cylinder’s frontal plane. Thin dashed lines represent 50% chance, error bars indicate *SEM*.

**Figure 4.**
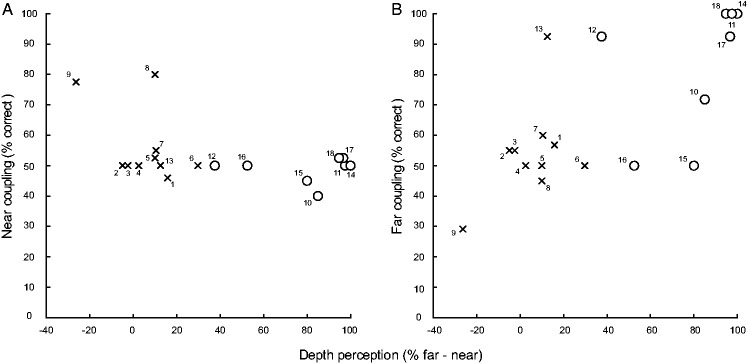
Scatter Plot of Coupling Versus Depth Perception. The percentage of correct coupling for (A) near and (B) far is plotted against depth perception represented by performance in percentage on far-defined minus near-defined stimulus for stereoscopic (open circles, *n *=* *8) and stereovision-impaired subjects (crosses, *n *=* *10), as defined by the individual baseline tests. Numbers correspond to individual subjects.

The stereoscopic subjects showed expected behavior (Figure 3A), based on the model proposed by Klink et al. (2009). The perceptual coupling is not different from chance level (50%) for the disparity-defined half cylinder in the near situation (black solid bar; *t*(7) = −1.05, *p *=* *.33), but significantly above chance level for the far-defined half cylinder (solid white bar; *t*(7) = 4.10, *p * < * *.01). The difference between performance in case of near versus far-defined disparity was significant, *t*(7) = 4.65, *p * < * *.01. The stereovision-impaired subjects performed differently than expected (Figure 3B) and showed no performance significantly above chance level for the near-defined half cylinder (black solid bar; *t*(9) = 1.35, *p *=* *.21) or the far-defined half cylinder (solid white bar; *t*(9) = 0.77, *p *=* *.* *46). There was no statistical difference between performance in case of near versus far defined disparity for this group of stereovision-impaired subjects, *t*(9) = 0.21, *p *=* *.84. Figure 4 shows the correlation between the percentage of correct response for depth perception and (a) near and (b) far coupling. Here, *depth perception* is expressed as performance (in % responded convex) on far stimulus minus near stimulus (−4.6 and 4.6 arcmin disparity, respectively) from the baseline experiment (see Figure 2). The % coupling on the *y*-axis reflects correct reporting of the motion direction of the full cylinder’s frontal plane from the motion coupling experiment (see Figure 3). For stereovision-impaired subjects, both the near and far-coupling plot do not suggest any correlation with depth perception. The near-coupling for stereoscopic subjects shows the same pattern. Far-coupling, however, seems more correlated with depth perception. Based on linear regression, we calculated a best fit for far coupling in the stereoscopic group (Figure 4B, open circles; *r *=* *0.42; *SSE *=* *0.28; *df *=* *6).

Two stereovision-impaired subjects stand out in the scatter plot for near coupling (Figure 4A). Both show a near-coupling of about 80%, whereas all other subjects remain around chance level (50%). Generally, the stereoscopic subjects show higher depth perception values and far coupling values. However, also in the group with subjects classified as stereoscopic based on the baseline test, we find subjects deviating from the rest of the group by showing low far-coupling (Figure 4B). One subject shows coupling around 50% and one around 70%, whereas the rest of the group shows performance levels higher than 90%. Another subject that was classified as stereoscopic shows a low depth perception value (40%).

## Discussion

We investigated perceptual coupling in stereoscopic and stereovision-impaired subjects using disambiguated half (inducer) cylinder and full bistable transparently rotating cylinders. Subjects were asked to report the rotation direction of the frontal plane of an ambiguous full cylinder projected coaxially to a rotating disparity-defined half cylinder. By providing stereoscopic depth information to the half cylinder we expected depth differentiated coupling in stereoscopic subjects, as reported by Klink et al. (2009). We hypothesized that stereovision-impaired subjects, that do not possess the ability to use stereoscopic depth cues to couple parts of objects, depend more on motion information for perceptual coupling and therefore show motion coupling between inducer and test stimulus.

First, we used results from our baseline test to classify subjects as stereoscopic or stereovision-impaired. For stereoscopic subjects, we found significant coupling for the frontal plane of the full ambiguous cylinder to the disparity-defined far half cylinder and coupling around chance level for the disparity-defined near half cylinder (Figure 3). The strong coupling in the far plane possibly reflects strong excitatory connections between neural representation of the disparity-defined half cylinder and the ambiguous full cylinder. The coupling around chance level between the full cylinder and the disparity-defined near half cylinder implies weak connections in the near field. These results are consistent with an occlusion-related coupling model presented in Klink et al. (2009); strong far coupling and weak near coupling. This neural network model explains perceptual coupling of kinetic depth stimuli in occlusion situations on the basis of lateral connections between similarly tuned neuronal clusters in the far depth plane. Those lateral connections facilitate sharing of spatially separated motion information. Integration of this global information helps resolve local ambiguities in the far depth plane and thereby the perception of partially occluded objects (Klink et al., 2009).

We expect stereovision-impaired subjects not to experience a difference between *near* and *far* half cylinder stimuli, so coupling should only be a function of the motion direction of the random pattern of the half cylinder. If the rotation direction of the ambiguous full cylinder in all trials would be coupled to the direction of the half cylinder, under our definitions, the percentage of coupling for the near stimulus would be 100% and for the far stimulus 0%, because we ask the subject to report the motion direction of the frontal plane of the ambiguous stimulus. A *correct* response for the motion direction of the frontal plane of the full cylinder corresponds with the opposite motion direction of the *far* half cylinder. However, our results show that on average stereovision-impaired subjects showed no significant motion coupling, with around 50% coupling for both depth fields (Figure3B), rejecting our hypothesis. Indeed, several studies show that individuals with binocular vision impairments, such as amblyopia or strabismus, experience difficulties with motion perception (Ho & Giaschi, 2007; Simmers et al., 2003; Wang et al., 2017). As it turned out from the etiology history of the subjects, all but two of the stereovision-impaired subjects reported to have experienced either amblyopia or strabismus in their early lives. This might be an explanation for the absence of motion coupling. Furthermore, details concerning medical history about visual anomalies were often undeliverable by the subjects, because of poor recollection. Because we do not possess information on the exact onset of the various causes underlying the stereovision impairment of all subjects, we cannot establish with certainty whether differences in performance among stereovision-impaired subjects can be explained by the period during which their vision became affected. Incomplete knowledge of underlying etiologies could interfere with finding support for our hypothesis. A quantitative test of amblyopia could be a valuable addition to this study.

Moreover, in our Method section, we made an important note on the suitability of the Stereo Fly Test. We used the Stereo Fly Test as a first indication of stereovision among subjects. However, The Stereo Fly Test assesses stereovision in the near plane, which means that passing this test could still mean subjects experience difficulties in the far plane. Furthermore, the sensitivity of this stereovision test is not high. It does not discriminate between stereo anomalies as defined by Richards (1970) and could yield false passes due to the use of nonstereoscopic cues (De La Cruz et al., 2016). Most subjects that passed the Stereo Fly Test typically also performed well in our baseline depth perception test. However, Subject 13 showed a rather large ω, indicating a broader range of disparity for the probability of reporting convex. This large ω, or inverse of slope, resembled more that of stereovision-impaired subjects. Statistical analysis based on Bayes factors indicated substantial evidence for this subject to be classified as stereovision-impaired (Figure 2B).

Besides monocular cues also binocular nonstereoscopic cues could be used to pass stereovision tests such as binocular luster, diplopia/confusion, binocular rivalry, and rivaldepth (Chopin et al., 2019). Although we cannot exclude the presence of all of these cues in our baseline stereovision test, we tested all our subjects for binocular rivalry. Most of our classified stereovision-impaired subjects reported seeing only one stimulus in the binocular rivalry test; however, two subjects experienced an overlapping percept of two stimuli. This might point to use of nonstereoscopic cues to identify depth, although their baseline test did not show any sign of this. All categorized stereoscopic subjects reported (alternating) dominance periods of one of both stimulus percepts. Since this experiment is limited and did not control for eye movements, we cannot exclude that our stereoscopic subject group contained subjects with specific stereo anomalies that would interfere with proper fixating such as strabismus. Our experiments could be improved by more extensively testing our subjects with stereo anomaly tests, such as those described by Ee and Richards (2002). Excluding all nonstereoscopic cues in a stereovision test is impossible, since disparity used to discriminate between stereoscopic and stereovision-impaired gives rise to such cues. But changing the task to order objects in depth instead of just detecting differences between depth objects could improve our baseline stereovision test, and thereby our classification of subjects into stereoscopic and stereovision-impaired (Chopin et al., 2019).

In our study of perceptual coupling, we found that stereoscopic subjects show strong motion coupling in the far plane, implying strong lateral connections between far depth neurons. The group of stereovision-impaired subjects showed no coupling, also not based on motion information. Although our baseline test formed a good indication for classifying subjects as stereoscopic or stereovision-impaired, it is still subject to arbitration. Taken together, our results point toward complexity and diversity among perceptual anomalies such as stereovision impairment.

## Data Availability

The datasets generated during and/or analyzed during the current study are available in the Donders Repository: https://WebDAV.data.donders.ru.nl/dcn/DAC_62001435_01_454/.
